# DNA damage, inflammation and aging: Insights from mice

**DOI:** 10.3389/fragi.2022.973781

**Published:** 2022-09-07

**Authors:** Ermioni S. Arvanitaki, Kalliopi Stratigi, George A. Garinis

**Affiliations:** ^1^ Department of Biology, University of Crete, Heraklion, Greece; ^2^ Foundation for Research and Technology-Hellas, Institute of Molecular Biology and Biotechnology, Heraklion, Greece

**Keywords:** DNA damage, DNA repair, inflammation, aging, diseases

## Abstract

Persistent DNA lesions build up with aging triggering inflammation, the body’s first line of immune defense strategy against foreign pathogens and irritants. Once established, DNA damage-driven inflammation takes on a momentum of its own, due to the amplification and feedback loops of the immune system leading to cellular malfunction, tissue degenerative changes and metabolic complications. Here, we discuss the use of murine models with inborn defects in genome maintenance and the DNA damage response for understanding how irreparable DNA lesions are functionally linked to innate immune signaling highlighting their relevance for developing novel therapeutic strategies against the premature onset of aging-associated diseases.

## Introduction

The growing list of syndromes associated with inborn defects in DNA repair and phenotypes resembling accelerated ageing points to genomic damage as a major culprit in the ageing process (Hoeijmakers, 1994). The gradual accumulation of persistent DNA lesions triggers the activation of potent inflammatory responses that are causal to age-related degeneration, metabolic abnormalities and cancer. Indeed, genome maintenance pathways and immune responses are highly conserved and tightly linked biological processes, supporting the notion that cells have encountered genotoxins and foreign pathogens nearly *ab initio*. For instance, induction of the SOS response, an inducible pathway governing DNA repair in bacteria affects their adaptation to antimicrobial tolerance, resistance, and virulence (Zgur-Bertok, 2013). In prokaryotes, the CRISPR-Cas system is also functionally linked to antiviral immunity and DNA repair (Babu et al., 2011). An efficient adaptive immune system in mammals requires that the non-homologous end-joining (NHEJ) pathway repairs the DNA strand breaks (DSBs) that occur during V(D)J recombination in developing T lymphocytes, ensuring the vast diversity of receptors necessary for overcoming pathogenic insults. (Boboila et al., 2012). Likewise, in B lymphocytes, an error-prone DNA repair mechanism is required to introduce a large number of nucleotide substitutions into a small area of the genome, in a process called somatic hypermutation of immunoglobulin variable genes, which produces variant antibodies with increased affinity for cognate antigens (Di Noia and Neuberger, 2007). Besides the functional contribution of DNA repair factors in the development and maturation of adaptive immunity, the distinct types of cells and tissues in mammals require that genome maintenance, DDR and innate immune responses, the organism’s natural response to harmful genotoxins, are tightly coordinated and timely mobilized [reviewed in (Ioannidou et al., 2016; Stratigi et al., 2017; Chatzidoukaki et al., 2020)].

## Linking DNA damage to innate immune signaling

There is ample evidence to suggest that genome instability drives aging and that DNA damage and DDR activation triggers a wide range of age-related phenotypes, including the activation of cell- and non-cell autonomous signaling pathways; the latter leads to the secretion of pro-inflammatory factors and the gradual infiltration of immune cells at sites of tissue damage with advancing age. DNA damage activates cytoplasmic NF-κB (nuclear factor kappa-light-chain-enhancer of activated B cells), an inducible transcription factor that translocates into the nucleus and functions as a pivotal mediator of inflammatory responses by binding to the DNA consensus sequence of target genes (Hayden and Ghosh, 2008). The interferon regulatory factors (IRFs) make up another family of immune-related transcription factors that become activated upon exposure to genotoxins. Induction of IRF-1 protein by either ionizing radiation or etoposide occurs through an increase in IRF-1 protein levels and an increase in the half-life of the IRF-1 protein; the response requires a functional DDR as cells defective in the DNA damage sensor ATM (ataxia telangiectasia, mutated) failed to increase IRF-1 in response to genotoxic stress (Pamment et al., 2002). IRF-3 and 5 provide further evidence that genome maintenance and the DDR are linked to the interferon signaling; IRF-3 is an *in vivo* target of DNA-PK (DNA-dependent protein kinase) (Karpova et al., 2002) that functions in the repair of DNA breaks by NHEJ and V(D)J recombination (Lieber et al., 2003) whereas IRF-5 is a direct transcriptional target of p53 upon exposure of cells to genotoxic insults (Mori et al., 2002). NKG2D also known as CD159 (Cluster of Differentiation 159) is an activating receptor that is predominantly expressed on the surface of cytotoxic immune cells. The presence of DNA lesions or stalled DNA replication forks triggers the upregulation of NKG2D ligands in mouse or human cells in an ATM-, ATR (Ataxia telangiectasia and Rad3 related)- or Chk1-dependent manner, allowing the immune system to detect and selectively remove damaged cells (Gasser et al., 2005; Gasser and Raulet, 2006; Champsaur and Lanier, 2010). DNAM-1 (DNAX Accessory Molecule-1) is a 65 kDa transmembrane glycoprotein that is constitutively expressed in the majority of T cells, NK cells, and macrophages (Shibuya et al., 1996). Low doses of chemotherapeutic genotoxins trigger the expression of DNAM-1 ligands in multiple myeloma cells in an ATM/ATR-dependent manner (Soriani et al., 2009). Likewise, ICAM-1 (Intercellular Adhesion Molecule 1), a transmembrane glycoprotein that promotes the adhesion of leucocytes to inflamed vascular endothelium, is also induced in response to ionizing irradiation in a p53-dependnent manner (Gaugler et al., 1997). Furthermore, in humans, the expression of all TLR (Toll-Like Receptor) genes, *TLR1* to *TLR10*, in blood lymphocytes and alveolar macrophages is induced by DNA damage with considerable inter-individual variability (Menendez et al., 2011). UV-irradiated keratinocytes form large cytoplasmic complexes, called “inflammasomes” to trigger the maturation, activation and secretion of pro-inflammatory cytokines (Faustin and Reed, 2008; Schroder and Tschopp, 2010).

DNA damage-induced inflammation can be both beneficial and detrimental for organismal survival. In higher organisms, the inflammatory response has evolved as an acute defense mechanism to eliminate the harmful irritant and allow the body to heal. With prolonged stimuli, however, as it is when DNA damage gradually accrues over time in cells, it leads to the activation of persistent DDR-mediated pro-inflammatory signals leading to systemic chronic inflammation, tissue degeneration and malfunction with old age. Cellular senescence is a great example of how irreparable DNA lesions underlie the persistent activation of innate immune signaling (Rodier et al., 2009; Campisi and d'Adda di Fagagna, 2007; Fumagalli and d'Adda di Fagagna, 2009). Senescent cells with chromatin hallmarks of irreparable DSBs secrete a wide range of senescence-associated secretory phenotype (SASP) factors, including inflammatory cytokines and chemokines as well as growth factors and extracellular matrix remodeling enzymes (Coppe et al., 2008; Ohanna et al., 2011; Acosta et al., 2013; Malaquin et al., 2013). SASP factors impinge on cell-fate decisions in neighboring cells or facilitate angiogenesis and promote the growth, invasion, and metastasis of tumor cells (Grivennikov et al., 2010). In support, older individuals show an increase in systemic inflammation as evidenced by the elevated levels of pro-inflammatory cytokines e.g., IL-6, clotting factors and acute phase reactants (Ferrucci et al., 1999; Cohen et al., 2003; Cavanagh et al., 2012; Shaw et al., 2013). Thus, there is an immense need to establish reliable *in vivo* animal models for understanding how DNA damage-driven inflammatory responses lead to some of the most challenging degenerative disorders of our time, as well as to develop rationalized therapeutic intervention strategies against chronic inflammation with aging.

## Mouse models for DNA damage-driven inflammation

Owing to their congenital defects in genome maintenance, DNA repair-deficient animals are valid models to test for the physiological relevance of DNA damage-driven inflammation in premature disease onset during normal and accelerated aging (Supplementary Table S1) (van de Ven et al., 2007; Schumacher et al., 2008a; Schumacher et al., 2008b; Garinis, 2008; Garinis et al., 2008; Garinis et al., 2009; Schumacher et al., 2009; Karakasilioti et al., 2013; Karakasilioti and Garinis, 2014; Ioannidou et al., 2016; Chatzidoukaki et al., 2020). Mice with engineered mutations in NER genes reliably mimic most of the pleiotropic and heterogeneous pathological symptoms seen in NER syndromes (Hasty et al., 2003; Niedernhofer et al., 2006; van der Pluijm et al., 2007; Schumacher et al., 2008a; Schumacher et al., 2008b; Garinis, 2008; Rieckher et al., 2021). ERCC1-XPF is a heterodimeric, structure-specific endonuclease complex required for lesion excision in nucleotide excision repair (NER) (Kamileri et al., 2012; Apostolou et al., 2019) that plays an analogous role in the repair of highly cytotoxic DNA inter-strand crosslinks (ICLs) (Niedernhofer et al., 2004). The *Ercc1* ^*−/−*^ and *Ercc1* ^-/Δ7^ mouse models of the human progeroid syndrome XFE (Niedernhofer et al., 2006; Gregg et al., 2012; Schermer et al., 2013; Pieren et al., 2021) provide compelling evidence that the persistent activation of DNA damage-driven innate immune responses lead to tissue-degenerative alterations. For instance, aP2*-Ercc1* ^*F/-*^ mice that carry the NER defect only in the white adipose tissue, rapidly accumulate DNA damage in adipocytes and manifest a chronic auto-inflammatory response leading to severe fat depletion and metabolic abnormalities (Karakasilioti et al., 2013). In aP2-*Ercc1F* ^*/-*^ mice, the fat depots manifest hallmarks of persistent DDR together with the transcriptional de-repression of pro-inflammatory factors, the infiltration of activated macrophages as well as the release of DAMPs known to initiate and perpetuate immune responses (Karakasilioti et al., 2013). In line with the contributing role of DNA damage-driven inflammation in the premature onset of age-related diseases, pharmacologic inhibition of NF-κB was shown to delay several pathological symptoms in progeroid ERCC1-defective mice (Tilstra et al., 2012). When the ERCC1 defect is restricted in macrophages in Lys2-*Ercc1* ^*F/-*^ mice, the tissue-infiltrating *Ercc1* ^*−/−*^ macrophages release extracellular vesicles whose cargo is targeted to diverse recipient cells leading to systemic metabolic reprogramming associated with enhanced cellular glucose uptake, increased oxygen consumption, chronic inflammation and overt pathology (Goulielmaki et al., 2020). More recently, the gradual accumulation of persistent DNA damage in hematopoietic cells of Vav-iCre^+/-^; *Ercc1* ^F/-^ mice led to an accelerated aging of the immune system associated with the progressive reduction of lymphocytes (Yousefzadeh et al., 2021); in turn, the aged immune system of Vav-iCre^+/-^; *Ercc1* ^F/-^ mice led to senescence and loss of tissue homeostasis in non-lymphoid organs. R-loops are nucleic acid structures composed of an RNA–DNA hybrid and a displaced single-stranded DNA that may spontaneously lead to DNA breaks (Skourti-Stathaki et al., 2011; Skourti-Stathaki and Proudfoot, 2014; Goulielmaki et al., 2021). In progeroid *Ercc1* ^*−/−*^ and naturally aged pancreata, DNA damage triggers the formation of R-loops leading to the release and build-up of single-stranded (ss)DNA fragments in the cytoplasm of cells stimulating a viral-like immune response (Chatzidoukaki et al., 2021). Owing to the great similarity of phenotypic features between *Ercc1* ^*−/−*^ and *Xpg* ^−/−^ animals mimicking the progeroid Cockayne syndrome (Barnhoorn et al., 2014), it is attractive to speculate that the latter animals would respond similarly in terms of the improper activation of the immune system. *Csb* ^*m/m*^ mice carrying an inborn defect in CSB involved in transcription-coupled (TC) NER present with lung inflammation and thrombogenic responses upon exposure to ozone, a well-established toxic environmental factor (Kooter et al., 2008). Interestingly, enhanced inflammation has also been noticed in *Xpa* ^*−/−*^ (Xeroderma Pigmentosum) mice that are defective in both the TC-NER and the global genome repair sub-pathways of NER (Miyauchi-Hashimoto et al., 1996). Loss of myelin and Purkinje cell death in *Csa* ^−/−^/*Xpa* ^−/−^ mice were accompanied by microglia and astrocyte activation and vascular inflammation in the brain, mirroring the neuronal dysfunction observed in XP patients (Kajitani et al., 2021). Similar to *aP2-Ercc1* ^*F/-*^ mice, increased cellular senescence in the adipose tissue of 4-weeks old *pol* η^−/−^ (DNA polymerase η) mice carrying a defect in lesion bypass polymerase was accompanied by the senescence-associated secretion of pro-inflammatory cytokines IL-6 and TNF-α (tumor necrosis factor α) (Chen et al., 2015). Besides DNA lesions associated with NER or post-replication repair machinery, inflammation is also triggered by irreparable modifications to DNA bases, including oxidation, alkylation and deamination. For instance, mice deficient in MBD4, a glycosylase involved in the detection and repair of deamination of methyl-cytosines, manifest greater colon inflammation and tissue injury leading to an increase in tumor burden (Yu et al., 2016). In line, alkyladenine DNA glycosylase and two members from the AlkB family of DNA repair enzymes (ALKBH2, and ALKBH3) are shown to protect against tissue injury and tumorigenesis in an inducible mouse model of inflammation-driven colon cancer (Meira et al., 2008; Calvo et al., 2012). 8-Oxoguanine glycosylase (OGG1) is the most prominent DNA glycosylase that is responsible for the removal of 8-OH-dG, an oxidative DNA lesion that is frequently formed in the DNA of aerobic organisms (Dianov et al., 1998). Importantly, OGG1–deficient macrophages are more apoptotic and inflammatory compared to wild type controls. Moreover, a defect in OGG1 leads to an increase in inflammasome activity and the premature onset of atherosclerosis in mice that are deficient for low-density lipoprotein receptor (Tumurkhuu et al., 2016). Naturally occurring RNA-DNA hybrids are frequently formed on promoters or termination regions when a nascent RNA molecule is hybridized with the DNA template before the two strands of the DNA duplex reanneal, leaving the non-template DNA single-stranded (Skourti-Stathaki et al., 2011; Skourti-Stathaki and Proudfoot, 2014). Persistent R-loops expose long stretches of ssDNA, leading to the spontaneous formation of DSBs or to transcription-associated mutagenesis (Muers, 2011; Wimberly et al., 2013; Costantino and Koshland, 2015). Partial loss‐of‐function biallelic mutations in RNase H2 genes, an endogenous endoribonuclease that cleaves the RNA strand in RNA-DNA duplexes, are the major cause of the autoinflammatory disorder Aicardi-Goutières syndrome (Crow and Manel, 2015). Likewise, a hypomorphic RNase H2 mouse model for Aicardi-Goutières syndrome associates with the induction of interferon-stimulated gene transcripts, a response that is dependent on the cGAS‐STING nucleic acid‐sensing pathway (Mackenzie et al., 2016). Mice with intestinal ablation of RNase H2, present with local intestinal inflammation, tissue damage and colorectal cancer (Aden et al., 2019). Werner syndrome is a premature aging disorder caused by mutations in a DNA helicase/exonuclease. Mice lacking the helicase domain of this protein exhibit an increase in serum inflammatory cytokines and metabolic abnormalities (Aumailley et al., 2015) that closely resembles the low grade, age-related inflammatory phenotype seen in Werner syndrome patients (Goto et al., 2012). Loss-of-function mutations in any of the Fanconi anemia (FA) genes, involved in DNA replication and the DDR, lead to neuronal decline, bone marrow failure, cancer, and premature aging (Bogliolo and Surralles, 2015). FANCC is required for antiviral host defenses and for the suppressing of inflammasome activation in mice, by removing damaged mitochondria (Sumpter et al., 2016); however, the mitophagy function of FANCC is independent of its role in DNA damage repair. Besides defects in genome maintenance, mice with mutations in genes coding for kinases involved in DDR manifest prominent inflammatory features. For instance, heterozygous mutant animals for *Smg1*, coding for a kinase with a known role in nonsense-mediated mRNA decay and the DDR, predisposes mice to higher levels of IL-6, CSF-1 and IL-1β cytokines and chronic inflammation in the lung and kidneys (Roberts et al., 2013). Mice deficient in ataxia telangiectasia mutated gene (ATM), upon exposure to lipopolysaccharide stimulation, associate with a prominent inflammatory phenotype that leads to Purkinje cell death (Yang et al., 2014). Likewise, *Atm* ^*−/−*^ mice are more sensitive to the deleterious effects of chronic dextran sulfate sodium-induced inflammation with greater upregulation of inflammatory cytokines, and higher percentages of activated CD69^+^ and CD44^+^ T-cells in the peripheral blood throughout treatment (Westbrook and Schiestl, 2010). Mice defective in DNA-PK, a nuclear DNA-dependent serine/threonine protein kinase involved in NHEJ and the DDR associate with an activation of cGAS-mediated antiviral innate immunity (Sun et al., 2020); DNA-PK binds DNA in the cytoplasm and stimulates the transcription of type I interferon (IFN), cytokine and chemokine genes in primary fibroblasts and mice (Ferguson et al., 2012). These findings could well explain why most patients with mutations in DNA-PKcs manifest a hyperactivated innate immune response and suffer from autoimmune diseases.

**FIGURE 1:**
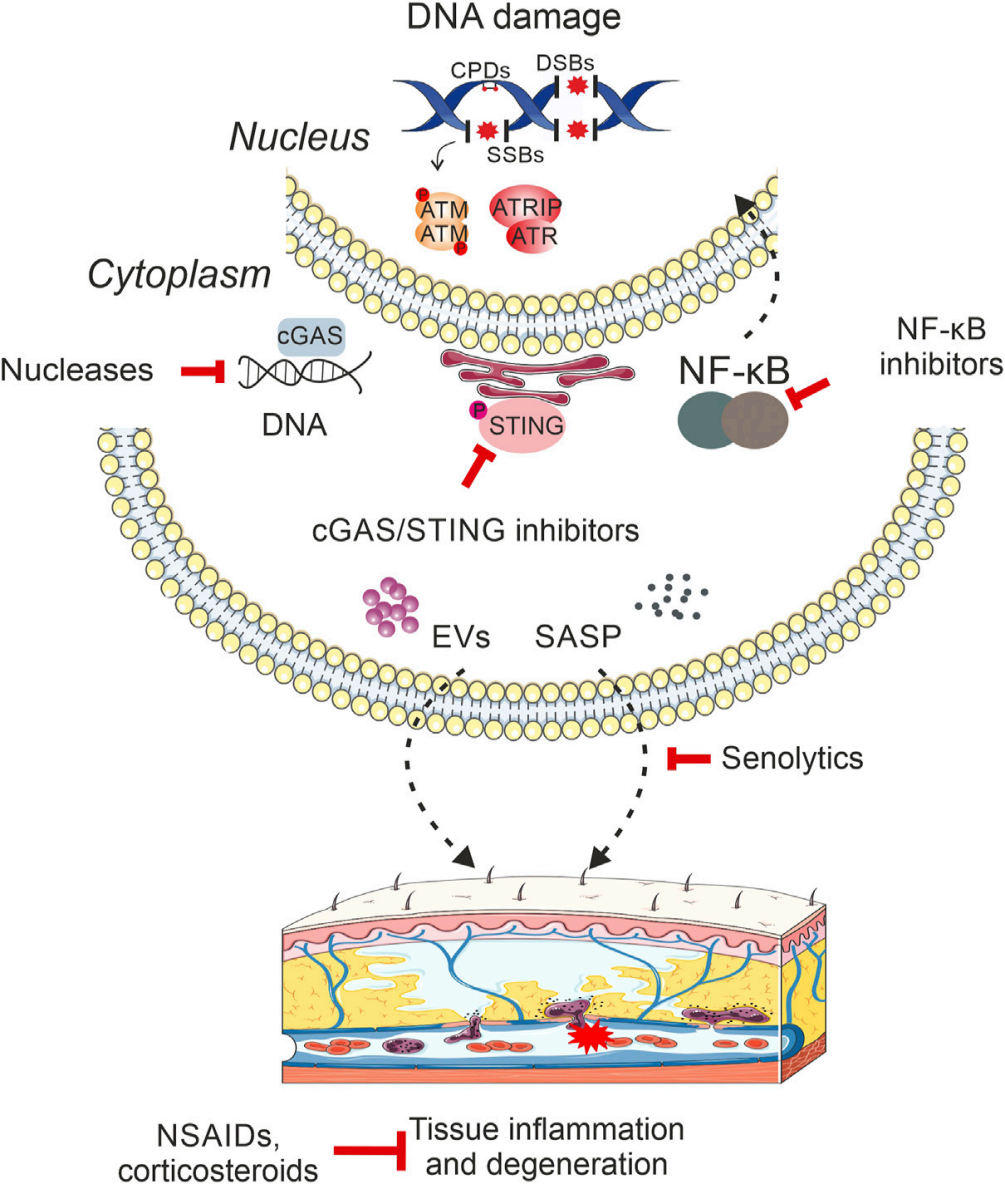
DNA damage-driven inflammation drives age-related pathology. DNA damage triggers the activation of innate immune responses, which act as a key mediator of tissue degenerative changes and age-related organismal decline. In particular, the presence of DNA breaks, small base modifications or bulky DNA adducts in the mammalian genome triggers the release of DNA moieties in the cytoplasm, which are predominantly sensed by the cGAS/STING signaling pathway stimulating the type I interferon (IFN) response. DNA damage also triggers the activation of NF-κB in the cytoplasm that translocates into the nucleus to activate the transcription of immune response gene targets. Moreover, the accumulation of irreparable DNA lesions may lead to the release of senescence-associated secretory phenotype (SASP) factors, such as cytokines, soluble growth factors, proteases as well as insoluble extracellular matrix components in the surrounding milieu. Cells with compromised genome integrity are also known to secrete extracellular vesicles that target a wide range of recipient cells leading to metabolic reprogramming and systemic inflammation. Eventually, the multiple links between persistent DNA damage and activation of innate immune responses in mammals lead to chronic inflammation that drives tissue deterioration, malfunction and organismal decline with aging. The recent development of rationalized intervention strategies (nucleases, corticosteroids etc.) against direct products of DNA damage itself i.e., nucleic acids or against inflammatory mediators are expected to profoundly lessen the adverse consequences of degenerative features that manifest with advancing age. Intervention methods targeting DNA damage-induced inflammatory stimuli are depicted in red inhibitory arrows.

In addition to genetic models with DNA repair/DDR defects, there is a wide range of genotoxins that are known to trigger a pro-inflammatory response in mice. For instance, exposure of the skin to ultraviolet (UV) light leads to the formation of cyclobutane pyrimidine dimers (CPDs) and pyrimidine (6-4) pyrimidone photoproducts (6-4PPs). The UV-induced photolesions interfere with the process of mRNA synthesis and DNA replication (Garinis et al., 2005) triggering skin inflammation and erythema, edema, and hyperplasia. Photolyases are enzymes that are capable of removing the UV-induced CPDs or 6-4PPs upon exposure to visible light (Garinis et al., 2006). However, these enzymes are not present in placental mammals. Using transgenic mice that express a marsupial (*Potorous tridactylis*) CPD-specific photolyase transgene, either ubiquitously or specifically in the basal keratinocytes of the epidermis, it was shown that light-dependent removal of CPDs (but not 6-4PPs) is the primary cause of the great majority of UV-exposed skin semi-acute responses, including sunburn, apoptosis, hyperplasia as well as non-melanoma skin cancer (Jans et al., 2005; Jans et al., 2006). Murine models of whole thorax or hemithorax irradiation reliably recapitulate the pathogenesis and the clinical symptoms of DNA damage-induced pneumonitis and fibrosis (Wirsdorfer and Jendrossek, 2017). Similar symptoms are also quickly developed in mice exposed to the radiomimetic DNA damaging chemotherapeutic drug Bleomycin (Moeller et al., 2008). Bacterial genotoxins are also known to trigger single- or double strand breaks (DSBs) in target host cells, leading to chronic inflammation that may ultimately facilitate the oncogenic processes. For instance, the enterotoxigenic *Bacteroides fragilis* secretes *B. fragilis* toxin, a zinc-dependent metalloprotease that can induce colitis and colorectal cancer in multiple intestinal neoplasia (Min) mice (Housseau and Sears, 2010). Likewise, mice infected with *Helicobacter hepaticus* associate with the presence of the bacterial genotoxin cytolethal distending toxin, a chronic inflammatory response and the development of hepatic dysplastic nodules (Ge et al., 2007). Regulatory T cells (Tregs) are a subpopulation of T cells that suppress the induction and proliferation of effector T cells. A recent study in Tregs derived from individuals with autoimmune diseases or from an animal model for multiple sclerosis revealed an enhanced DDR signaling in these cells resulting from an increase in mitochondrial oxidative stress and impaired lysosomal function (Alissafi et al., 2020). Thus, as inflammation further inflicts DNA damage upon immune-system cells in the periphery, DNA damage-driven innate immune responses may take on a momentum of their own due to the amplification and feedback loops of the immune system leading to severe degenerative tissue changes over time (Figure 1).

## Rationalized therapeutic strategies against DNA damage-driven inflammation

As DNA damage drives inflammation and aging, it is tempting to consider therapeutic strategies that would lessen the inflammatory burden and delay the premature onset of associated tissue degenerative changes with old age. Non-steroidal anti-inflammatory drugs (NSAIDs) are medicines that are widely used to reduce inflammation and relieve symptoms of long-term pain. However, the use of NSAIDs is often associated with gastrointestinal problems, including stomach irritation and reflux and may increase the risk of cardiovascular conditions (Vonkeman and van de Laar, 2010). Corticosteroids present another category of anti-inflammatory drugs that are typically used to treat rheumatologic and chronic inflammatory diseases that associate with aging. However, depending on the dose, type of steroid and length of treatment, corticosteroid drugs may trigger a wide range of side effects, including weight gain, muscle weakness and lower resistance to infection, osteoporosis, diabetes, stomach irritation etc., (Yasir et al., 2022). Moreover, the high prevalence of chronic inflammatory diseases and the pervasive risks associated with the long-term use of available anti-inflammatory drugs requires that a new class of therapeutic regimens becomes available that would avoid the unnecessary use of NSAIDs and corticosteroid drugs. This is particularly relevant for the treatment of chronic inflammatory conditions that require repetitive or continuous treatment regimens. The use of senolytic drugs that can selectively eliminate senescent cells (and by inference also the release of pro-inflammatory SASP factors) or the targeting of the SASP response itself (rather than the senescent cells) may serve as an alternative way to reduce the inflammatory load driven by irreparable DNA damage (Gasek et al., 2021). Importantly, senolytics may not need to be administered continuously to trigger senolysis, thereby minimizing potential harmful side effects. However, the SASP response often entails hundreds of proteins and non-protein signaling factors, whose composition depends on the cell type involved and the mechanism driving senescence (Birch and Gil, 2020). Moreover, in certain cell types, such as in macrophages, the accumulation of age-related biomarkers e.g., lipofuscin and β-galactosidase or the increase in the mRNA levels of senescent-associated factors p16INK4A, p21CIP1 or SASP-associated IL-6 and IL-8 protein levels (Goulielmaki et al., 2020) may only reflect a reversible response of these cells to physiological stimuli (Hall et al., 2017), rather than the premature onset of replicative or stress-induced senescence (Franceschi et al., 2000). For DNA damage-driven inflammatory responses, it is, therefore, valid to consider alternative options that would target the cause rather than the symptoms of inflammation. Owing to the fact that DNA damage triggers the accumulation of cytoplasmic DNA moieties or RNA-DNA hybrids in cells (Chatzidoukaki et al., 2021), there have been recent attempts to develop a new class of therapeutic regimens that may prove valuable in ameliorating the harmful effects of DNA damage-driven inflammation. For instance, cytoplasmic DNA species are rapidly sensed by the enzyme cGAS (cyclic GMP-AMP synthase), which in turn activates the adaptor protein STING (stimulator of interferon genes) on the endoplasmic reticulum. As the cGAS-STING pathway can sense the cytosolic DNA activating the innate immune response, selective small molecules were developed as inhibitors, with the potential to target the cGAS-STING axis in humans. However, inhibition of the cGAS-STING pathway may provoke adverse effects in humans by increasing susceptibility to infection (Decout et al., 2021). Likewise, several immunosuppressant drugs that may be used to combat DNA damage-driven inflammatory responses by inhibiting e.g., JAK/STAT or NF-κB signalling, various TLRs, NADPH oxidase, IL-1β and TNF-α associate with an increased risk of infection, as well as with akinetic mutism, toxic encephalopathy, seizures, tremor and neurobehavioral changes (Fireman et al., 2004). More recently, we reasoned that the removal of cytoplasmic ss DNA moieties or RNA-DNA hybrids from cells would reduce the inflammatory load in damaged cells *in vivo*. To test this, we used extracellular vesicles (EVs) to deliver recombinant Mung Bean S1 or RNase H1 nucleases to inflamed DNA repair-deficient cells that rapidly accumulate DNA damage or to wild-type cells previously treated with a genotoxin. Interestingly, the nuclease-mediated removal of cytosolic nucleic acids from these cells curbed the Type I IFN response ameliorating the DNA damage-induced phenotype in targeted cells (Chatzidoukaki et al., 2021).

## Conclusions

There is much work to be done before we will be able to dissect the functional links between persistent DNA damage and inflammation *in vivo*. The use of progeroid murine models with tissue-specific defects in genome maintenance will allow us to further delineate the causal contribution of specific cell types to systemic inflammation with old age (Figure 1). In parallel, animal models with tagged DNA repair factors coupled to functional genomics and proteomics strategies may prove valuable for identifying new gene targets or protein partners that could link genome maintenance with innate immune signaling. It will also be essential to identify how an active DDR originating from any alterations in the physicochemical structure of the DNA activates cytoplasmic stress responses and the release of proinflammatory factors in the tissue microenvironment. Likewise, it will be vital to dissect the functional links between DNA damage-driven chronic inflammation and metabolic rewiring with old age. Finally, the recent development of novel therapeutic strategies indicates that, in the long run, it may be more valuable to invest in approaches targeting the DNA damage itself rather than suppressing downstream proinflammatory signals. Such strategies could open new, meaningful avenues towards the development of new rationalized therapeutic interventions against a wide range of adverse pathological outcomes during aging (Tilstra et al., 2012; Karakasilioti et al., 2013).
