# Supplementary material for: DNA damage, inflammation and aging: Insights from mice
Source: Front Aging. 2022 Sep 7;3:973781. doi: 10.3389/fragi.2022.973781 (PMC9490123; doi:10.3389/fragi.2022.973781)
Supplement: Supplementary file 1 [file DataSheet1.PDF]

**Table 1. A list of animal models with relevance to DNA damage-driven inflammation.**

NER: Nucleotide Excision repair, ICL: Inter-strand Crosslink Repair, DSBR: Double Strand DNA Repair, BER: Base Excision Repair, PRR: Post-Replication Repair, FA: Fanconi Anemia. Inh: Inhibitor.

| Mouse Model | DNA Repair | Mechanism | References |
|---|---|---|---|
| *Ercc1* $^{-/-}$ | NER, ICL, DSBR | Cytosolic ssDNAs | [60] |
| *Ercc1* $^{-/\Delta 7}$ | NER, ICL, DSBR | - | [51, 52] |
| aP2-*Ercc1* $^{F/-}$ | NER, ICL, DSBR | Pro-inflammatory factors, DAMPs | [43] |
| Lys2-*Ercc1* $^{F/-}$ | NER, ICL, DSBR | Metabolic reprogramming, EV secretion | [55] |
| Vav-iCre$^{+/-}$; Ercc1 $^{F/-}$ | NER, ICL, DSBR | mTOR activity | [56] |
| *Xpa* $^{-/-}$ | NER | Enhanced local immunosuppression | [63] |
| *Csb* $^{m/m}$ | NER | Cytosolic ssDNAs | [60] |
| *Xpc* $^{-/-}$ | NER | Cytosolic ssDNAs | [60] |
| *Csa* $^{-/-}$ *Xpa* $^{-/-}$ | NER | Activated NF-κB, vascular inflammation | [64] |
| *pol η* $^{-/-}$ | PRR | Senescence-associated secretory phenotype | [65] |
| *Mbd4* $^{-/-}$ | BER | - | [66] |
| *Alkbh2* $^{-/-}$, *Alkbh3* $^{-/-}$ | BER | - | [67], [68] |
| *Ogg1* $^{-/-}$ *Ldlr* $^{-/-}$ | BER | Oxidized mtDNA in the cytosol of *Ogg1* -/- macrophages | [70] |
| *Rnaseh2b* $^{A174T/A174T}$ | RER | Cytosolic RNA:DNA heteroduplexes | [75] |
| *H2b* ΔIEC | RER | p53 activity | [76] |
| *Wrn* Δhel/Δhel, *Wrn* $^{-/-}$ | BER, DSBR | Increase in ROS, protein oxidation, defect in autophagy | [77] |
| *Fancc* $^{-/-}$, *Fanca* $^{-/-}$ | FA | Pro-inflammatory cytokines, Notch signaling | [79], [80] |
| *Smg1* $^{+/gt}$ | DDR | Elevated tissue cytokines, oxidative damage | [81] |
| *Atm* $^{-/-}$ | DDR | Activated microglia and astrocytes | [82] |
| *Atm* $^{-/-}$ | DDR | Oxidative stress and persistent immune response | [83] |
| **Treatment** | **DNA Repair** | **Mechanism** | |
| DNA-PK inh. Nu7441 | DSBR | cGAS activation | [84] |
| UV light | NER | DNA replication and mRNA synthesis inhibition | [86] |
| Thorax IR | DSBR | DAMP release from epithelial and endothelial lung cells | [90] |
| Bleomycin | DSBR | Ionizing radiation imitant | [91] |
| *Bact. fragilis* (*Apc* $^{+/-}$ mice) | DSBR | Bact. fragilis toxin - zinc-dependent metalloprotease | [92] |
| *Hel. hepaticus* | DSBR | Cytolethal distending toxin | [93] |
